# Analysis of the Relationship between Type II Diabetes Mellitus and Parkinson's Disease: A Systematic Review

**DOI:** 10.1155/2019/4951379

**Published:** 2019-11-23

**Authors:** Fauze Camargo Maluf, David Feder, Alzira Alves de Siqueira Carvalho

**Affiliations:** ^1^Medical Student of Centro Universitario Saude ABC, Centro Universitario Saude ABC, FMABC, Santo Andre 09060-870, Brazil; ^2^Department of Pharmacology, Centro Universitario Saude ABC, FMABC, Santo Andre 09060-870, Brazil; ^3^Department of Neurosciences, Centro Universitario Saude ABC, FMABC, Santo Andre 09060-870, Brazil

## Abstract

In the early sixties, a discussion started regarding the association between Parkinson's disease (PD) and type II diabetes mellitus (T2DM). Today, this potential relationship is still a matter of debate. This review aims to analyze both diseases concerning causal relationships and treatments. A total of 104 articles were found, and studies on animal and “in vitro” models showed that T2DM causes neurological alterations that may be associated with PD, such as deregulation of the dopaminergic system, a decrease in the expression of peroxisome proliferator-activated receptor-gamma coactivator-1*α* (*PGC-1α*), an increase in the expression of phosphoprotein enriched in diabetes/phosphoprotein enriched in astrocytes 15 (PED/PEA-15), and neuroinflammation, as well as acceleration of the formation of alpha-synuclein amyloid fibrils. In addition, clinical studies described that Parkinson's symptoms were notably worse after the onset of T2DM, and seven deregulated genes were identified in the DNA of T2DM and PD patients. Regarding treatment, the action of antidiabetic drugs, especially incretin mimetic agents, seems to confer certain degree of neuroprotection to PD patients. In conclusion, the available evidence on the interaction between T2DM and PD justifies more robust clinical trials exploring this interaction especially the clinical management of patients with both conditions.

## 1. Introduction

The prevalence of type 2 diabetes mellitus (T2DM) is 370 million people in the world. The T2DM is most frequent in adulthood; however, in the last years, the prevalence of T2DM is increasing in adolescents and children [1, 2]. T2DM is a chronic metabolic disease characterized by long-term insulin resistance and a decrease of *β*-cell function and population. These factors impair insulin release and consequently cause hyperglycemia [3, 4]. However, genetic and environmental factors are responsible for 20% of *β*-cell failure in the diabetic population [2].

One of the consequences of chronic diabetes is the production of toxic aggregates of the islet amyloid polypeptide (IAPP). The IAPP might contribute to *β*-cell dysfunction [5].

Parkinson's disease (PD) affects about 1% of people over 65 and up to 4-5% of people over 85, and thus it represents the second most common neurodegenerative disorder [6, 7].

The diagnosis of PD is still based on the presence of symptoms and clinical signs such as typical asymmetric manifestation, the most common finding being tremor at rest in the upper limbs associated with bradykinesia, rigidity, and gait difficulty [8].

The etiology of PD is based on the combination of genetic (10% of cases) and probably environmental factors [9]. Moreover, most of PD cases are idiopathic, and the exact etiology is still unclear [7].

The concept of protein misfolding disorder (PMD) is relevant for the understanding of the potential association of T2DM and PD. Protein misfolding disorders (PMD) are diseases caused by a protein or peptide that has been misfolded, aggregated, and accumulated in certain tissues. There are at least 30 different PMDs, including neurodegenerative, systemic, and metabolic disorders such as PD and T2DM [10].

The specific protein that misfolds and could contribute to the pathogenesis of T2DM is called amylin. This protein is deposited as aggregates and has been found in the pancreas and brain tissue [5].

Human alpha-synuclein is the protein that misfolds and is thought by many to be related to the development of PD [11]. It is encoded by the alpha-synuclein gene (*SNCA*) and is expressed in neurological tissues and extraneurological tissues such as the pancreas [11]. The *SNCA* gene is associated with glucose and insulin regulation through K-channel modulation in *β* cells of the pancreas [12, 13] responsible for aggregation, toxicity, and Ca^2+^ binding [14].

In PD, there is the aggregation, deposition, and dysfunction of alpha-synuclein, which causes the accumulation and propagation of alpha-synuclein to various brain regions and cellular populations [15]. The levels of alpha-synuclein depend on the proportion between the synthesis, aggregation, and clearance of alpha-synuclein. A dysfunction in this proportion may result in high levels of alpha-synuclein that might favor the formation of toxic species [16]. Therefore, it is not unlikely that the two proteins, amylin and alpha-synuclein, would affect each other *in vivo*, causing T2DM and PD [17]. In addition, recent discoveries have showed common pathways that probably relate neurodegenerative mechanisms with abnormal glucose metabolism and abnormal mitochondrial function [18].

The association between T2DM and PD was previously reported since patients with T2DM seem to have an increased risk of also developing PD [19]. In a large cohort of 8 million people, Pablo-Fernandez et al., [20] showed a higher rate of subsequent PD following T2DM. However, there are studies that showed the opposite or no relation between these diseases [21, 22].

Another point suggesting a possible correlation between PD and T2DM has been demonstrated in the interaction of hypoglycemic and antiparkinsonian drugs. The recent studies took into account the effects of some drugs used to treat PD, such as levodopa, which induces both hyperglycemia and hyperinsulinemia [23], whereas others (including the ergot dopamine agonist bromocriptine) may increase insulin sensitivity [24]. In addition, hypoglycemic drugs such as biguanide [25], sulfonylureas [26], thiazolidinediones [27], and incretinomimetics [28] were used in the management of patients with PD.

T2DM and PD are common diseases that negatively affect patients' quality of life. Thus, it is of utmost importance not only to study these diseases in an isolated manner but also to investigate their correlations and interactions. So, this review aims to assess the risk factor association, the genetic link and the pathophysiologic interactions between T2DM and PD. Moreover, this review intends to determine the modifications on the clinical features when these diseases are associated and to evaluate the impact of hypoglycemic drugs on PD and antiparkinsonian drugs on T2DM.

## 2. Materials and Methods

The review was based on the analysis of studies retrieved via PubMed up to September, 2019. Articles were screened according to the following eligibility criteria: original articles describing the relationship between PD and T2DM, unrelated to any other disease, using *in vivo* (human and animal) and *in vitro* models. The descriptors using the MeSH database were as follows: “(“Parkinson Disease”[Mesh]) AND “Diabetes Mellitus”[Mesh],” “(“Parkinson Disease”[Mesh]) AND “Insulin Resistance”[Mesh],” “(“alpha-Synuclein”[Mesh]) AND “Diabetes Mellitus”[Mesh],” “(“alpha-Synuclein”[Mesh]) AND “Insulin Resistance”[Mesh],” “(“Islet Amyloid Polypeptide”[Mesh]) AND “Parkinson Disease”[Mesh],” “(“Metformin”[Mesh]) AND “Parkinson Disease”[Mesh],” “(“Sulfonylurea Compounds”[Mesh]) AND “Parkinson Disease”[Mesh],” “(“Thiazolidinediones”[Mesh:NoExp]) AND “Parkinson Disease”[Mesh],” “(“Glucagon-Like Peptide 1”[Mesh]) AND “Parkinson Disease”[Mesh],” “(“Bromocriptine”[Mesh:NoExp]) AND “Diabetes Mellitus”[Mesh],” “(“Exenatide”[Mesh:NoExp]) AND “Parkinsonian Disorders”[Mesh],” “(“Levodopa”[Mesh]) AND “Diabetes Mellitus”[Mesh],” “(“Dipeptidyl-Peptidase IV Inhibitors”[Mesh]) AND “Parkinson Disease”[Mesh]”, “(“Sodium-Glucose Transporter 2 Inhibitors”[Mesh]) AND “Parkinson Disease”[Mesh].” Letters, reviews, and articles in languages other than English were excluded.

## 3. Results

**Figure 1:**
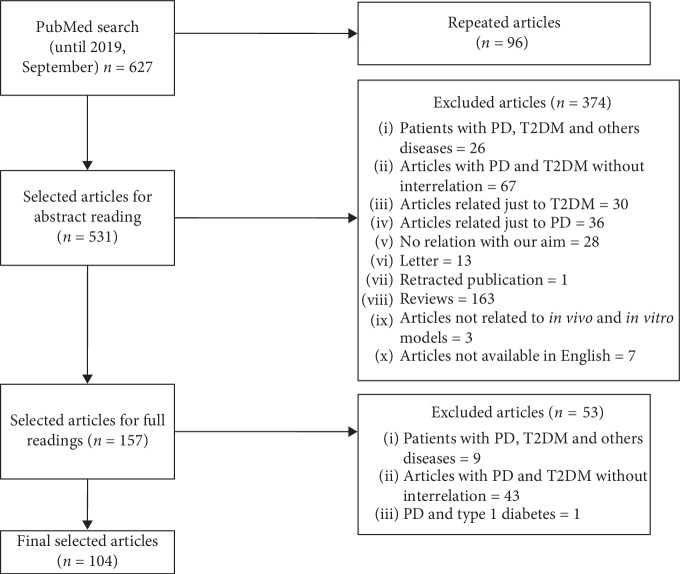
Flow diagram of literature search to identify articles evaluating the relationship between T2DM and PD.

A total of 627 articles were found (Table 1). Among them, 96 were duplicated in searches with different descriptors, leaving 531 abstracts to be evaluated. Then, 157 articles were set aside to be fully read, from which 104 were used in the bibliography (Figure 1).

Our study showed connections between PD and T2DM in relation to epidemiology (Table 2), genetics (Table 3), clinical manifestations (Table 4) and treatment (Tables 5 and 6). We also found a relationship between both conditions in the pathophysiological mechanisms. In PD, mitochondrial dysfunction [29], mutations in different genes encoding alpha-synuclein, PINK-1 (PTEN-induced putative kinase 1), and DJ-1 (Protein deglycase) may favor the development of T2DM [12, 30]. Similarly, pathways of T2DM may influence the development of PD such as metabolic inflammation [31], downregulation of dopamine in the nigrostriatal pathway [32–34], long-term hyperglycemia [35], decrease in the expression of *PGC-1α* (peroxisome proliferator-activated receptor-gamma coactivator-1*α*) [36–39], increase in the expression of PED/PEA-15 (phosphoprotein enriched in diabetes/phosphoprotein enriched in astrocytes 15 protein) [40], increased methylglyoxal levels [41, 42], and the formation of alpha-synuclein amyloid fibrils [17].

## 4. Discussion

### 4.1. Type 2 Diabetes vs. Parkinson's Disease: Epidemiology

The prevalence of T2DM patients suffering from PD is slightly heterogeneous, ranging between 3.4 and 9.1% [123], whereas in the general population the prevalence of PD is 1-2 per 1000 [124]. In this review, several studies suggested that T2DM might increase the risk of developing PD [19, 20, 43–49]. This might possibly be explained due to the fact that both diseases share common pathophysiological pathways, such as increased iron levels [125, 126] that may be involved in the insulin regulation in the nigrostriatal pathway [32] and low expression of *PGC-1α* gene that could lead to mitochondrial dysfunction [18, 29]. However, there are some studies that supported an inverse association [50–54] or the lack of association [22, 55–57] between them. These conflicting findings could possibly be explained by factors like self-reported T2DM diagnosis [55] and study design (case-control studies) [22, 50–54] when the temporal relationship between disease onset and exposure is not clear. Additionally, a recent cohort with a sample size of approximately 8 million subjects indicated T2DM as a risk factor for PD [20]. This study excluded patients with cerebrovascular disease and drug-induced and vascular Parkinsonism which were not considered in previous studies [20].

As for the inverse association, PD patients less frequently suffer from T2DM owing to the decrease in sympathetic activity caused by PD [59] and the use of L-dopa, a drug that increases glycogenolysis and inhibits the use of peripheral glucose [58, 59, 127]. Only one study showed a higher chance for the development of T2DM in PD patients [60]. A possible explanation for this is the abnormal tolerance to glucose in approximately half of the patients with PD [128] that could evolve to T2DM.

Many factors related to lifestyle and genetics are interconnected with the risks of T2DM and PD. Advanced age is the main risk factor for developing PD [129] and an important factor for the onset of T2DM [130]. Interestingly, it was observed that smoking increased the risk of T2DM and reduced the risk of PD [131].

### 4.2. Type 2 Diabetes vs. Parkinson's Disease: Genetics

PD and T2DM are complex, multifactorial disorders with a combination of environmental and genetic factors involved in the pathogenesis of the diseases. PD and T2DM with genetic alterations represent 5–10% of cases [132].

The genetic relationship between these diseases was confirmed by genetic mapping of the genes in both diseases. In this study, a genome-wide association study (GWAS) and microarrays showed 478 genes closely associated with confirmed PD and T2DM [63]. In a different study, using only GWAS, 84 PD, and T2DM-associated genes were identified [62]. Therefore, it is believed that genes associated with T2DM can be used to identify PD genes, and the PD genes can identify T2DM genes, as well [63]. However, in another study, the GWAS analysis of PD and T2DM did not reveal any significant relationship between the diseases [61]. This fact could be explained because the authors exclusively analyzed the top candidate variants which precluded to find rare genetic variants or copy-number variations [61].

Moreover, T2DM and PD patients have a common haplogroup, B5b [64], indicating that they share same genetic mutations in mitochondrial DNA (mtDNA), such as the presence of the adenine in position 709 of the mtDNA (709G>A). The mitochondrial dysfunction present in both pathologies could therefore be explained by this finding [77, 133].

Finally, in microarray analyses, seven deregulated genes, the amyloid precursor protein (*APP*) gene in particular, were quantified by gene expression from blood samples of T2DM and PD patients [63]. Actually, the expression of *APP* is increased in PD and prediabetic patients [134, 135], suggesting that high levels of the protein encoded by this gene in the blood of T2DM patients could be an indicator of neurodegeneration [63, 136].

### 4.3. Type 2 Diabetes vs. Parkinson's Disease: Clinical Manifestations

Patients with both T2DM and PD have a noticeable aggravation of motor symptoms, higher degree of cognitive impairment and earlier onset of complications [65–69, 71]. Regarding motor symptoms, the worsening was especially observed in the postural instability and mobility of these patients [69–71], whereas attention impairment and slower speed of thinking were noted in the cognitive processes [65, 67]. Such damages may be associated with dopamine regulation deficiency and neuroinflammation [31–34], confirmed by image tests that showed white matter injuries, lacunar infarctions and cortical atrophy in T2DM patients [137, 138]. Moreover, motor complications (motor fluctuations and dyskinesia) happened one year earlier compared with subjects with only PD [68]. Therefore, longer periods of hospitalization, as well as daily care, are typically required for these patients [72].

In addition, the risk of cerebrovascular accident (CVA) in patients with T2DM and PD is higher than in those with T2DM only [72]. A possible reason for this higher incidence is the fact that PD patients have increased levels of homocysteine due to the use of L-dopa increasing the synthesis of free radicals and enhancing neuroinflammation [139].

Patients with PD and T2DM showed a reduction in HbA1c and an improvement in the lipid profile [72]. The use of drugs for the treatment of PD, bromocriptine in particular, as well as the reduction in the sympathetic activity and hypothalamic-pituitary-adrenal axis impairment [59] would probably decrease the production of catecholamines and cortisol resulting in lower levels of glycemia [59]. Concerning the improvement in the lipid profile, no relevant hypothesis has yet been made. One possible explanation is the fact that dyskinesia and decreased physical activity observed in some PD patients may lead to less food intake, which could reduce the lipid level [140]. However, more studies are necessary to explore the potential pathophysiology of better levels of lipid in PD and T2DM patients.

### 4.4. Type 2 Diabetes vs. Parkinson's Disease: Pathophysiology

**Figure 2:**
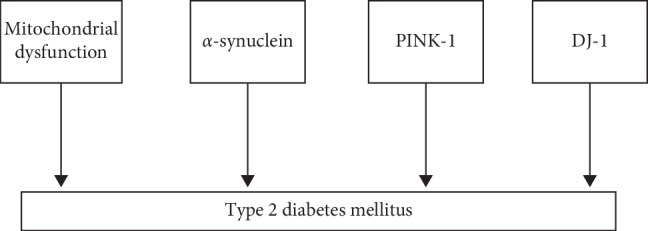
Pathophysiological mechanism of PD that may favor the development of T2DM.

**Figure 3:**
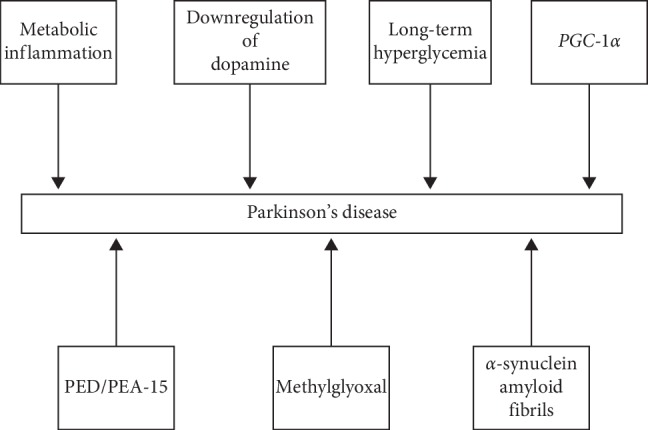
Pathophysiological mechanism of T2DM that may favor the development of PD.

Based on results of “in vitro” studies and studies using animal models, this review suggests that there are common pathophysiological features involving T2DM and PD (Figures 2 and 3).

Many studies suggested pathophysiological mechanisms already related to PD that may favor the development of T2DM such as mitochondrial dysfunction [29], mutations in genes encoding alpha-synuclein, PINK-1 (PTEN-induced putative kinase 1), and DJ-1 (Protein deglycase) [12, 30].

The neuroinflammatory processes observed in PD activate microglia cells, causing the overproduction of reactive oxygen species (ROS) and proinflammatory cytokines (nitrous oxide and tumor necrosis factor-alpha) with resulting mitochondrial dysfunction [141].

To simulate the mitochondrial dysfunction in PD, many studies used the neurotoxin 1-methyl-4-phenyl-1,2,3,6-tetrahydropyridine (MPTP). This drug mimics PD-like symptoms and inhibits complex I of the respiratory chain [142–144] by decreasing ATP production and triggering the release of free radicals, thus leading to the death of dopaminergic neurons [29]. The mitochondrial dysfunction caused by PD may accelerate the progression of insulin resistance via increased production of ROS [145]. Therefore, it is possible that the mitochondrial dysfunction observed in PD could promote the development of T2DM.

In relation to mutations in gene-encoding proteins, studies using alpha-synuclein in rats showed that this protein lowers the resistance to insulin [12]. In PD, the mutated alpha-synuclein could trigger the formation of aggregates of the protein [146], thus impairing insulin resistance and increasing the likelihood of developing T2DM [147].

Moreover, the deficiency of DJ-1 inhibits the aggregation of alpha-synuclein and increases the resistance to insulin in rats [148]. Likewise, PINK-1 deficiency, present in PD, also favors resistance to insulin [30]. Hence, mutations of alpha-synuclein, DJ-1, and PINK-1 are important related factors that may favor the development of T2DM in PD patients.

On the other hand, we also found pathophysiological components of T2DM that could lead to PD such as metabolic inflammation [31], downregulation of dopamine in the nigrostriatal pathway [32–34], long-term hyperglycemia condition [35], decrease in the expression of *PGC-1α* (peroxisome proliferator-activated receptor-gamma coactivator-1*α*) [36–39], increase in the expression of PED/PEA-15 (phosphoprotein enriched in diabetes/phosphoprotein enriched in astrocytes 15 protein) [40], increased methylglyoxal levels [41, 42], and the formation of alpha-synuclein amyloid fibrils [17].

Many studies evaluated the use of MPTP in diabetic rats to analyze the pathophysiological interaction between both diseases. The use of MPTP in diabetic rats resulted not only an accelerated loss of dopaminergic neurons and the activation of glial cells in the substantia nigra but also an increase in the activation of inflammatory molecules, including NLRP3 and alpha-synuclein aggregates in the pancreas and in the brain. In addition, the endoplasmic reticulum stress markers CHOP and GRP78 were positively regulated in the pancreas, liver, and brain of mice with T2DM [31]. Therefore, the metabolic inflammation in T2DM may contribute to the occurrence of PD.

Futhermore, it was observed that insulin regulates dopamine synthesis and uptake within the substantia nigra [34]. Some studies have revealed that the impaired insulin signaling in T2DM causes the degeneration of the nigrostriatal dopaminergic pathway and an exacerbated neurodegeneration in animals [32–34] that consequently could facilitate the onset of PD-like symptoms. In addition, long-term hyperglycemia in a rat model also caused nigrostriatal dopaminergic neurodegeneration due to elevated basal oxidative burden and motor impairments that are similar to early parkinsonian symptomatology [35].

In insulin resistance, the gene *PGC-1α*, a regulator of enzymes involved in mitochondrial respiration, shows reduced expression [36, 39]. Additionally, *PGC-1α* is repressed by PARIS (ZNF746), a protein that causes neurodegeneration in PD due to parkin inactivation [37]. Many researchers observed that *PGC-1α* protects against the destruction of dopaminergic neurons [37, 38]. As a consequence, the decreased protection of these neurons in T2DM may lead to the development of PD.

Another pathophysiological mechanism in T2DM is the protein PED/PEA-15. This protein is increased in many cells (skeletal muscles, adipocytes, skin fibroblasts, and peripheral blood leukocytes) in T2DM patients. A recent study showed that rats overexpressing this protein had a reduction in dopaminergic activity, a fact that may induce the development of PD [40].

One recent association between PD and T2DM is the ADTIQ (1-acetyl-6,7-dihydroxy-1,2,3,4-tetrahydroisoquinoline) and its precursor methylglyoxal (a subproduct of glucose metabolism that is increased in diabetic patients) [149]. ADTIQ was recently discovered in frozen human PD brain tissues [150], and its role could be as an endogenous neurotoxin that causes PD [151]. In diabetic rats, it was observed that the accumulation of ADTIQ, produced by the reaction of dopamine and methylglyoxal, caused neuronal injury, oxidative stress, and apoptosis [41, 42]. Therefore, it is possible that ADTIQ may be an important factor to increase the risk of PD in patients with T2DM.

Finally, the formation of alpha-synuclein aggregates can be observed in PD, whereas pancreatic amyloid plaques formed by the pancreatic islet amyloid polypeptide (IAPP) are present in T2DM. In one study, it was noted that the speed of alpha-synuclein aggregation is higher when it interacts with IAPP [17], thus showing an increased propensity for the development of PD in T2DM cases. A possible explanation could be the excessive glycation process in T2DM patients. In this process, proteins undergo posttranslational modification, which affects the alpha-synuclein structure and increases its aggregation [152].

### 4.5. Type 2 Diabetes vs. Parkinson's Disease: Treatment Related Issues

#### 4.5.1. Antidiabetic Drugs

Biguanides (metformin) activate the AMP-activated kinase protein (AMPK) [153] responsible for the homeostatic control of cellular energy balance, glucose absorption in the muscle, and the inhibition of hepatic glucose production [154]. In rodent models, these properties neutralize the toxicity of MPTP through the reduction of oxidative stress levels [155], the neurogenic potential of this drug [156], and the restoration of the mitochondrial membrane potential [89]. Metformin also was associated with neuroprotection by ameriolating the neurotoxicity of alpha-synuclein in human neuroblastoma SH-SY5Y cells [157]. Futhermore, two studies observed the neuroprotection of metformin through the improvement in the motor function of the animals [25, 84, 86]. Nevertheless, one study showed an increased risk of PD associated with the use of metformin in a mouse model [85]. In human trials, metformin was usually combined with other antidiabetic drugs such as sulphonylureas [47] and thiazolidinediones [117]. Only one trial studied metformin individually and found a higher incidence of PD [88]. The increased risk of PD could be explained by the elevation of AMP/ATP and ADP/ATP ratios that occurs through the activation of AMPK, with inhibition of the mitochondrial complex I and, as a result, an increase in vulnerability as well as degeneration of dopaminergic neurons [85]. The effect of metformin in patients with PD has not been fully elucidated. Therefore, the potential benefit or harm of metformin in patients with PD remains to be determined.

Sulphonylureas (glibenclamide, tolbutamine, and glipizide) stimulate insulin release by inhibiting the ATP-sensitive K+ (K ATP) channel of pancreatic beta-cells resulting in the closure of the potassium channels and opening of calcium channels [158]. However, these channels are present not only in the pancreas but also in cardiac, skeletal cells, and in neurons of the central nervous system (cortex, basal ganglia, hippocampus, hypothalamus, and striated muscles in particular) [159, 160]. The activation of K ATP channels in the central nervous system has already been associated as a protector of mitochondria function [161]. Consequently, it is possible that the inhibition of K ATP channels might intensify mitochondria dysfunction and aggravates the neurological complications in PD patients [108]. Sulphonylureas did not show neuroprotection in any of the analyzed studies [26, 47, 107, 108], which could be a predisposing factor for the development of PD.

Several studies investigated the effect of thiazolidinediones (pioglitazone and rosiglitazone) in the treatment of PD [28, 109–122]. They act on peroxisome proliferator-activated receptor-gamma (PPAR-y) receptors by boosting the action of insulin [162] and bind to the protein of the external mitochondrial membrane [163–165]. This interaction showed positive effects on the activity of complex I of the respiratory chain in neuronal cells, which can reverse mitochondrial dysfunction in PD [166]. Besides, these drugs showed a protective action against neurodegeneration and neuroinflammation in MPTP-treated rodents, triggered by either a lipopolysaccharide model or an L-dopa-induced dyskinesia model, as well as in humans [28, 109–122]. This protective property of thiazolidinediones can be explained by the activation of PPAR-y, which reduced or reversed the microglial polarity, resulting in a decrease in nitric oxide synthase (NOS) activity, oxidative stress, and free radical release [13, 115, 116]. Some studies, however, suggested the beneficial action of thiazolidinediones through the inhibition of monoamine oxidase B (MAO-B) preventing the breakdown of dopamine and, consequently, increasing the levels of dopamine [113]. Surprisingly, a recent 44-week placebo-controlled phase 2 study in 210 PD patients did not show the neuroprotective effect of this drug [119]. In addition, a different study could not identify a reduction in the biomarkers of PD in patients on pioglitazone, such as leukocyte *PGC-1α*, plasma interleukin 6, and urine 8-hydroxydeoxyguanosine [120]. However, it is important to underline that these biomarkers are not FDA approved. Therefore, according to these new studies, there appears to be no potential for this drug to treat PD neurodegeneration.

Incretin mimetics drugs (GLP-1/GIP agonists and DPP-4 inhibitors) activate glucagon-like peptide-1 (GLP-1) or glucose-dependent insulinotropic polypeptide (GIP) receptors on pancreatic beta-cells stimulating insulin secretion and synthesis [100, 166]. The activation of the GLP-1 receptor by GLP-1 agonists (exenatide, liraglutide, and semaglutide) seems to prevent the death of dopaminergic neurons and improve motor and cognitive functions [27, 90–97, 99–101, 167, 168]. These facts can be explained by the increase in levels of tyrosine hydroxylase and vesicular monoamine transporter 2 (VMAT-2) in neurons of the nigrostriatal system along with the inhibition of microglial activation and the release of proinflammatory mediators [91, 95–97]. A recent study suggested the possible neuroprotective effect of exenatide through the activation of protein kinase B (PKB) and the mitogen-activated protein kinase (MAP kinase) pathways. Together, they influence not only neuroinflammation but also neuronal and mitochondrial survival pathways [169]. Besides, a groundbreaking randomised, double-blind, placebo-controlled trial demonstrated that PD patients treated with exenatide once in a week for 48 weeks had a 3.5-point advantage over placebo in the Movement Disorders Society Unified Parkinson's Disease Rating Scale (MDS-UPDRS) [97]. In fact, this is the first time that a disease-modifying drug for the diabetes treatment had a relevant positive effect on PD progression [170]. Moreover, another study reported that the neuroprotection offered by exenatide persisted after 12 months of treatment in 20 patients with PD [98], thus providing positive evidence for the potential of GLP-1 agonists. Regarding GIP agonists (D-Ala2-GIP-glu-PAL), neuroprotection in MPTP-treated rats and cell cultures was confirmed through the reduction in dopaminergic neurons and an increase in the antiapoptotic protein Bcl-2 (*β*-cell lymphoma 2), which prevented apoptosis and reduced chronic brain inflammation [100, 102, 103]. Finally, DPP-4 inhibitors slow degradation of GLP-1 increasing the insulin secretion [171]. This drug has demonstrated antiparkinsonian effects [104–106] explained by the reduction in ROS expression, brain mitochondrial dysfunction in diabetic rats [104], and the suppression of neuroinflammatory and apoptotic cascades in models of PD induction in rats [105]. Moreover, a nationwide case-control study showed, for the first time, a decreased risk of future PD in patients using DPP-4 inhibitors [106]. However, the whole mechanism of action of DPP-4 against neurodegeneration in PD is not fully understood [172]. Hence, further studies on the neuroprotective potential of incretin mimetics for the treatment of PD should be conducted.

#### 4.5.2. Antiparkinsonian Drugs

A relationship between T2DM and L-dopa therapy [71, 73] and dopaminergic agonists, especially bromocriptine [74, 76, 77, 80–83], has been detected. In trials with rodents, L-dopa therapy caused a decrease in insulin secretion in glucose tolerance tests due to the dopamine increase in pancreatic cells [173]. In addition, a partial loss of L-dopa efficacy in PD patients who developed T2DM [71] was observed probably because some pathophysiological mechanisms of T2DM can aggravate PD. Bromocriptine has an inhibitory effect on the production and release of prolactin, preventing disorders of carbohydrate and lipid metabolism due to the excessive amount of this hormone. In animals and patients with T2DM, there is an improvement in glucose intolerance [75, 78, 81, 174, 175], a reduction in the production of hepatic glucose, in serum lipid levels [54, 76, 80, 83] and in the risk of cardiovascular complications [77].

## 5. Conclusion

“In vitro” and animal studies suggest that T2DM causes neurological alterations that may be associated with PD, such as deregulation of the dopaminergic system, a decrease in the expression of peroxisome proliferator-activated receptor-gamma coactivator-1*α* (*PGC-1α*), an increase in the expression of phosphoprotein enriched in diabetes/phosphoprotein enriched in astrocytes 15 (PED/PEA-15), and neuroinflammation, as well as acceleration of the formation of alpha-synuclein amyloid fibrils. Epidemiological studies suggested that T2DM increases the risks of PD. In addition, clinical studies described that Parkinson's symptoms were notably worse after the onset of T2DM. Regarding treatment, the action of antidiabetic drugs, especially incretin mimetic agents, seems to confer a certain degree of neuroprotection to PD patients. In conclusion, the available evidence on the interaction between T2DM and PD justifies more robust clinical trials exploring this interaction especially the clinical management of patients with both conditions.

## Figures and Tables

**Table 1:** Relations of descriptors and studies found in PubMed database.

Descriptors	Number of articles
Parkinson's disease × diabetes mellitus	335
Parkinson's disease × insulin resistance	39
Alpha-synuclein × diabetes mellitus	19
Alpha-synuclein × insulin resistance	5
Islet amyloid polypeptide × Parkinson's disease	5
Metformin × Parkinson's disease	9
Sulfonylurea × Parkinson's disease	16
Thiazolidinediones × Parkinson's disease	19
GLP-1 × Parkinson's disease	7
Bromocriptine × diabetes mellitus	98
Exenatide × Parkinson's disease	19
Levodopa × diabetes mellitus	54
Dipeptidyl-peptidase IV inhibitors × Parkinson's disease	2
Sodium-glucose transporter 2 inhibitors × Parkinson's disease	0

**Table 2:** Trials correlating PD and T2DM as a risk factor.

Risk of PD in patients with T2DM	Authors	Sample
Increased (*n* = 9)	De Pablo-Fernandez et al. [20]	T2DM: 2,017,115 Controls: 6,173,208
	De Pablo-Fernandez et al. [43]	PD: 79 (14 with T2DM) Controls: 4.919 (842 with T2DM)
	Yang et al. [44]	T2DM: 36.294 (550 with PD) Controls: 108.882 (1232 with PD)
	Yue et al. [45]	PD: 6441 Controls: 1.755.191
	Sun et al. [46]	T2DM: 603.413 (1.613 with PD) Controls: 472.718 (809 with PD)
	Wahlqvist et al. [47]	T2DM: 64.166 Controls: 698.587
	Schernhammer [48]	PD: 1.931 (126 with T2DM) Controls: 9.651 (482 with T2DM)
	Xu et al. [49]	T2DM: 21.611 (172 with PD) Controls: 267.051 (1393 with PD)
	Hu et al. [19]	T2DM: 1.098 (24 with PD) Controls: 50.454 (609 with PD)

Decreased (*n* = 5)	Miyake et al. [50]	PD: 249 (10 with T2DM) Controls: 368 (39 with T2DM)
	D′ Amelio et al. [51]	PD: 318 (13 with T2DM) Controls: 318 (31 with T2DM)
	Leibson et al. [52]	PD: 197 (18 with T2DM) Controls: 197 (24 with T2DM)
	Powers et al. [53]	PD: 352 (26 with T2DM) Controls: 484 (61 with T2DM)
	Herishanu et al. [54]	PD: 93 (11 with T2DM) Controls: 93 (26 with T2DM)

Not related (*n* = 4)	Savica et al. [22]	PD: 196 (13 with T2DM) Controls: 196 (17 with T2DM)
	Palacios et al. [55]	PD: 656 Controls: 147,440
	Driver et al. [56]	PD: 556 Controls: 21.285
	Simon et al. [57]	PD: 530 (37 with T2DM) Controls: 171.349 (3.722 with T2DM)

Risk of T2DM in patients with PD		
Decreased (*n* = 2)	Becker et al. [58]	PD: 3.637 (291 with T2DM) Controls: 3.637 (308 with T2DM)
	Scigliano et al. [59]	PD: 178 (6 with T2DM) Controls: 534 (58 with T2DM)
Increased (*n* = 1)	Pressley et al. [60]	PD: 791 (235 with T2DM) Controls: 24.040 (5.175 with T2DM)
Total = 21		

**Table 3:** Trials correlating genetic profile and T2DM/PD.

Authors	Sample (controls/PD/T2DM)	Correlation between T2DM and PD
Chung et al. [61]	500/500/102	No correlation
Santiago et al. [62]	46/50/10	84 genes
Santiago et al. [63]	91/101/11	478 genes 7 genes (microarray)

**Table 4:** Trials correlating clinical features of patients with PD and T2DM.

Influence of T2DM on PD	Authors	PD with T2DM/PD without T2DM
Major cognitive impairment (*n* = 3)	Ong et al. [65]	PD with T2DM: 11 PD without T2DM: 51
	Petrou et al. [66]	PD with T2DM: 12 PD without T2DM: 24
	Bohnen et al. [67]	PD with T2DM: 15 PD without T2DM: 133

Worsening of motor symptoms and/or postural instability (*n* = 4)	Mohamed Ibrahim et al. [68]	PD with T2DM: 25 PD without T2DM: 25
	Pagano et al. [69]	PD with T2DM: 21 PD without T2DM: 51
	Kotagal et al. [70]	PD with T2DM: 13 PD without T2DM: 26
	Cereda et al. [71]	PD with T2DM: 466 PD without T2DM: 921

Influence of PD on T2DM		
Reduction of glycemia and/or glycated hemoglobin and lipid profile improvement (*n* = 1)	Scheuing et al. [72]	PD with T2DM: 1579 PD without T2DM: 177.413

**Table 5:** Protection of anti-Parkinson's drugs in T2DM.

LEVODOPA	BROMOCRPTINE
**No**	**Yes**
**Human trials**: Cereda et al. [71], Rosati et al. [73]	**Human trials** Chamarthi et al. [74] Roe et al. [75], Ghosh et al. [76], Gaziano et al. [77], Vinik et al. [78], Pijl et al. [79]
	**Animal studies:** Ezrokhi et al. [80], Luo et al. [81], Luo et al. [82], Cincotta et al. [83]

**Table 6:** Protection of antidiabetic drugs in PD.

METFORMIN		
**Yes**	**No**	**Yes**
**Animal studies**: Katila et al. [25], Ryu et al. [84], Ismael et al. [85], Patil et al. [86], Choi et al. [87]	**Human trials**: Kuan et al. [88]	**Cell culture studies**: Fitzgerald et al. [89]

GLP-1 Agonist (Exenatide/Liraglutide/Semaglutide**)**		
**Yes**	**Yes**	**Yes**
**Animal studies**: Zhang et al. [90], Bassil et al. [27], Cao et al. [91], Hansen et al. [92], Kim et al. [93], Li et al. [94], Bertilsson et al. [95], Harkavyi et al. [96]	**Human trials**: Athauda et al. [97], Bassil et al. [90], Aviles-Olmos et al. [98], Aviles-Olmos et al. [99]	**Cell culture studies**: Jalewa et al. [100], Perry et al. [101]

GIP Agonist (D-Ala2-GIP-glu-PAL)		
**Yes**		**Yes**
**Animal studies**: Feng et al. [102], Li et al. [103]		**Cell culture studies**: Jalewa et al. [100]
DPP-4 Inhibitors		
**Yes**	**Yes**	
**Animal studies**: Abdelsalam et al. [104], Pipatpiboon et al. [105]	**Human trials:** Svenningsson et al. [106]	

Sulphonylurea		
**No**	**No**	**No**
**Animal studies**: Obata et al. [26], Kou et al. [107]	**Human trials**: Wahlqvist et al. [47]	**Cell culture studies**: Tai et al. [108]

Thiazolidinediones		
**Yes**	**No consensus**	**Yes**
**Animal studies**: Pinto et al. [28], Martinez et al. [109] Ren et al. [110], Barbiero et al. [111], Pisanu et al. [112], Quinn et al. [113], Hunter et al. [114], Dehmer et al. [115], Breidert et al. [116]	**Human trials**: Brakedal et al. [117], Brauer et al. [118], Ninds Exploratory trials in Parkinson's disease FS-ZONE, Investigators et al. [119], Simon et al. [120]	**Cell culture studies**: Jung et al. [121], Xing et al. [122]
